# Transcription dynamics of heat shock proteins in response to thermal acclimation in *Ostrinia furnacalis*

**DOI:** 10.3389/fphys.2022.992293

**Published:** 2022-09-26

**Authors:** Yudong Quan, Zhenying Wang, Hongyi Wei, Kanglai He

**Affiliations:** ^1^ State Key Laboratory for Biology of Plant Diseases and Insect Pests, Institute of Plant Protection, Chinese Academy of Agricultural Sciences, Beijing, China; ^2^ College of Agronomy, Jiangxi Agricultural University, Nanchang, China

**Keywords:** heat shock proteins, thermal tolerance, heat shock response, *Ostrinia furnacalis*, extreme climate events

## Abstract

Acclimation to abiotic stress plays a critical role in insect adaption and evolution, particularly during extreme climate events. Heat shock proteins (HSPs) are evolutionarily conserved molecular chaperones caused by abiotic and biotic stressors. Understanding the relationship between thermal acclimation and the expression of specific HSPs is essential for addressing the functions of HSP families. This study investigated this issue using the Asian corn borer *Ostrinia furnacalis*, one of the most important corn pests in China. The transcription of *HSP* genes was induced in larvae exposed to 33°C. Thereafter, the larvae were exposed to 43°C, for 2 h, and then allowed to recover at 27 C for 0, 0.5, 1, 2, 4, 6, and 8 h. At the recovery times 0.5–4 h, most population tolerates less around 1–3 h than without recovery (at 0 h) suffering continuous heat stress (43 C). There is no difference in the heat tolerance at 6 h recovery, with similar transcriptional levels of *HSPs* as the control. However, a significant thermal tolerance was observed after 8 h of the recovery time, with a higher level of *HSP70*. In addition, the transcription of *HSP60* and *HSC70* (heat shock cognate protein 70) genes did not show a significant effect. *HSP70* or *HSP90* significantly upregulated within 1–2 h sustained heat stress (43 C) but declined at 6 h. Our findings revealed extreme thermal stress induced quick onset of *HSP70* or *HSP90* transcription. It could be interpreted as an adaptation to the drastic and rapid temperature variation. The thermal tolerance of larvae is significantly enhanced after 6 h of recovery and possibly regulated by *HSP70*.

## Introduction

Ongoing global climate change has caused a substantial increase in the occurrence of extreme thermal events (Meehl and Tebaldi, 2004; Alexander et al., 2006; IPCC, 2013; Ma et al., 2021). The phenotypic plasticity of ectotherms is a nongenetic strategic response to environmental variation, including thermal extremes (Hoffmann et al., 2013; Gunderson and Stillman, 2015; van Heerwaarden et al., 2016). Insects, as ectotherms, exhibit plasticity in a suite of traits related to thermal extremes, have fast generation times, and thus are considered good models to study plasticity (Sgrò et al., 2016; Gibert et al., 2019). Understanding plasticity has taken on increased importance in the context of rapid climate change as extreme events are more frequent and show increased variability (Fox et al., 2019). For example, insects living at higher latitudes have evolved plastic responses to survive in cold winters, but extreme events are increasingly disrupting the reliability of environmental signals (Sgrò et al., 2016; Gibert et al., 2019). Thermal extremes can lead to heat injury, as well as to a series of changes at the molecular, biochemical, and physiological levels in insects (Chown and Nicolson, 2004; Bowler, 2018; Ma et al., 2021), such as water content (Prange, 1996), cell membranes (Neven, 2000; Rensing and Ruoff, 2002), and activity of enzymes (Greenspan et al., 1980). Meanwhile, thermal acclimation of insects is a form of plasticity that enables organisms to adjust their physiology, following chronic or brief exposure to stressful stimuli (Bowler, 2005; Angilletta, 2009). The challenge is understanding how thermal extremes or stresses act on and manipulate acclimation in insects.

Heat shock proteins (HSPs) are known as stress molecular chaperones, which are essential for environmental adaptation and are associated with a wide range of physiological and biochemical processes (King and MacRae, 2015). According to their molecular size, the major families of HSPs are named HSP100, HSP90, HSP70, HSP60, and HSP40, and small proteins (sHSP). HSPs play a vital role in insects’ responses to extreme temperatures (Basha et al., 2012; King and MacRae, 2015), as well as responding to cold/heat tolerances (Rutherford and Lindquist, 1998), density, starvation, poison, ultraviolet-C, and diapause (Sosalegowda et al., 2010; Tedeschiab et al., 2015; Zhang et al., 2015; Tedeschi et al., 2016). HSP70 and HSP90 are highly conserved in all eukaryotes and prokaryotes and consist of two highly conserved domains: an N-terminal ATP-binding domain and a C-terminal substrate-binding domain (Lindquist. 1986; Taipale et al., 2010). These proteins generally serve in regulating the adaptions of insects to adverse environments and serve as a predominant self-protection mechanism (Chen et al., 2015a; Guo and Feng, 2018). The heat shock protein 70 family includes stress-inducible genes (HSP70s) and constitutively expressed members or heat shock cognates (HSC70s) (Daugarrd et al., 2007). HSC70s participate in various processes in an unstressed cell, such as folding of proteins after translation or membrane translocation, and may or may not be influenced by stress (Denlinger et al., 2001; Daugarrd et al., 2007). HSP60 helps protect against protein aggregation of denaturing proteins during diapause and operates the bending and assembling of enzymes and other protein complexes related to energy metabolism (Meyer et al., 2003; Brackley and Grantham, 2009; Mayer, 2010; King and MacRae, 2015).

There is an approximate doubling in the frequency and the magnitude of regional heat wave events from 1960 to 2018 in China, with the top three regional heat wave events in the summers of 2013, 2017, and 2003 (Wang and Yan, 2021). *Ostrinia furnacalis*, Lepidoptera (Crambidae), is one of the most common pests of corn in China, which causes economic losses in summer (Zhou and He, 1995; He et al., 2018). The survey showed that it detected about 300 larvae per 100 corn plants in Jilin Province and even 100% damage in Qinhuangdao city (Hebei province) (Yuan et al., 2013). On a summer day (from July to August), the temperature often climbs to 38 or more and has been reported to significantly affect the *O. furnacalis* population (Zhou et al., 2018).

The well-known mechanism used to cope with extreme temperatures is the expression of stress-inducible HSPs (Feder and Hofmann 1999; Sørensen et al., 2003; King and MacRae, 2015; Ma et al., 2021). The transcription of genes (mRNAs) encoding inducible heat shock proteins (in the response) appears to be temperature-sensitive in insects (Lindquist, 1986; Theodorakis and Morimoto, 1987; Morimoto, 1993; Sistonen et al., 1994; Prahlad and Morimoto, 2009; Lewis et al., 2016; Jin et al., 2020; Tian et al., 2020). However, the HSP expression of thermal tolerance or acclimation of *O. furnacalis* and the molecular mechanisms in its physiology, are poorly understood. In our study, we focused on the high temperatures that induce HSPs and then investigated *O. furnacalis* thermal tolerance in progressive recovery from extremely high temperatures. We related dynamic changes of potential heat shock protein genes through reverse-transcription quantitative polymerase chain reactions (RT-qPCRs). Our study helps improve the understanding of the mechanisms of thermotolerance in *O. furnacalis* and at a molecular level, analyzes the acclimation characteristics.

## Materials and methods

### Insects

Asian corn borer, *O. furnacalis,* adults were collected from Luoyang city, Henan province (111.8′112.59 “E, 33.35′35.05″ N). Progenies were maintained at insectary (27 ± 1°C, 70 ± 10% RH, and L16: D8 h) with standard artificial diet and techniques (Zhou and He, 1995) in the insectary to establish a laboratory colony. The 12-day-old (4th instar) larvae, which are more resilient to stress, were used in the following experiments.

### Heat shock and recovery experiments

Ten larvae were placed into a 10-ml centrifuge tube with 12 holes (0.2 cm in diameter). Three experiments for exposure to extremely high temperatures were carried out. Two independent replicates were performed in the experiments.

Exp. 1: the extreme heat wave in August has been reported at 43 C in some parts of China (https://news.bjd.com.cn/2022/08/13/10133544.shtml). Our previous findings have shown that *O. furnacalis* eggs could not survive at 45 C (Quan et al., 2022). Larvae (ca. 400) were exposed to extremely high temperatures of 43 C with 50% ± 10% RH (usually it is ∼40%–55% on 5–7 days at 35°C–40°C in the field) (Zhou et al., 1996; Chen et al., 2012) for 2 h in the chambers. Then, the larvae were transferred to 27°C, 70% ± 10% RH for recovery. After 0, 0.5, 1, 1.5, 2, 4, and 8 h, fifty larvae each were transferred to the chamber set up with a temperature of 43°C and 50% ± 10% RH. Survivors were checked by touching a larva’s head with a small paintbrush, and they recorded every hour until all larvae were dead. Another fifty larvae were maintained at 27°C (70% ± 10% RH); thus, unexperienced exposure to 43 C was used as control. They were also subjected to the chamber at 43°C and 50% ± 10% RH, and survivors were checked out hourly.

Exp. 2: to generate a time series of heat shocks, 30–40 larvae were exposed to treatment temperatures of 31, 33, and 35 C for 1, 2, 4, 6, and 8 h in a chamber (VM04/100, Heraeus, Germany) with 50% ± 10% RH. Treated larvae were then frozen in liquid nitrogen and stored at −80 C before being used for mRNA extraction. Larvae were maintained at 27°C, and RH 70% ± 10% were used as a control.

Exp. 3: to investigate the effect of recovery time, 270 larvae were first exposed to 43°C and 50% ± 10% RH for 2 h. They were then transferred to 27 C for recovery. Finally, 30 larvae at a time were frozen in liquid nitrogen and stored at −80 C after 0, 0.5, 1, 1.5, 2, 4, 8, 16, and 32 h of recovery.

Exp. 4: larvae were exposed to 43 C 50% ± 10% RH for 2 h. They were then transferred to 27°C and 70% ± 10% RH for recovery. After 0, 0.5, 1, 2, 4, 6, and 8 h, the larvae were re-exposed to 43°C and 50% ± 10% RH for 0, 0.5, 1, 1.5, 2, 4, 8, and 16 h. Lastly, fifty larvae at a time were immediately frozen in liquid nitrogen and stored at −80 C before being used for mRNA extraction.

### RNA isolation and cDNA synthesis

TRIzol (Invitrogen, United States) reagent was used to extract total RNA from sampled insects, following the manufacturer’s instructions. In brief, frozen tissues were smashed for ∼8 min (adding 15 ml liquid nitrogen per 2–3 min) by hand. Then, ∼0.1 g of insect powder was homogenized with 1 ml of TRIzol reagent into a 1.5-ml centrifuge tube and incubated at room temperature for 5 min. Phase separation of RNA was performed using 200 µl chloroform, 500 µl isopropanol for precipitation, washing with 800 μl, and 70% ethanol. The reaction was stopped by washing, followed by centrifugation at 4°C, 7,500 rpm for 5 min, and the supernatant was removed. We repeated the washing several times, subsequently air-dried samples, and then re-dissolved them (the final sediment) in RNase-free water. The purity and concentration of RNA were measured by agarose gel electrophoresis using a Nanodrop 2000 spectrophotometer (ThermoScientific, United States). The samples with an A260/280 ratio of ≥1.8 were used in downstream applications. The aliquots were frozen and stored at −80 C.

The cDNA synthesis was performed using a commercial reverse transcription kit (AT341-02, TransGen Biotech, China). An aliquot of RNA (2000 ng) from each sample was mixed with the reaction including 10 µl of the sample (dissolved by RNase-free water), 1 µl of anchored Oligo (dT) _18_ primer, 1 µl of the TransScript RT/RI enzyme mix, 1 µl of the gDNA remover, and 10 μl of the TS reaction mix. This solution was mixed; then, the reaction was incubated at 42 C for 30 min and 85 C for 5 s. When the reaction was stopped, the cDNA was stored at −20 C.

### Quantification of *HSP* gene expression (qRT-PCR)

According to the sequences of HSP60 (accession No: XM_028317890.1), HSC70 (accession No: JF708083.1), HSP70 (accession No: XM_028322992.1), and HSP90 (accession No: GU230734.1) in Asian corn borer registered in GenBank, the specific primers were designed. Through comparison and verification, 10 primers were used to amplify selected target genes (Supplementary Table S1).

The kit used for fluorescence quantitative real-time PCR (qRT-PCR) was SYBR^@^ Premix Ex Taq™ (TaKaRa). Ribosomal protein L8 (18sRNA) was used as a reference gene (Wang et al., 2007). The amplification program was set as follows: 1) initial denaturation 2 min at 95°C; 2) 95°C for 5 s and 60°C for 30 s; 3) step 2) was repeated for 40 cycles. Each sample was replicated three times, and we calculated the average value (Wang et al., 2007). The amplification efficiency of each heat shock protein gene was recorded, and the different heat shock protein genes related to transcription were analyzed by the 2^−△△CT^ method (Livak and Schmittgen, 2001).

### Data analysis

Larval thermal tolerance data, i.e., the survival as influenced by the time in the various recovery time treatments after exposure to 43°C, were analyzed and calculated by the Kaplan–Meier function. HSP expression levels under different temperatures were analyzed using a one-way analysis of variance (one-way ANOVA). The means were compared with Fisher’s protected LSD test, and statistical significance was considered at *p* < 0.05. All data analyses were processed in SPSS 17.0 software (Chicago: SPSS Inc.).

## Results

### Heat shock-induced thermal tolerance

**FIGURE 1:**
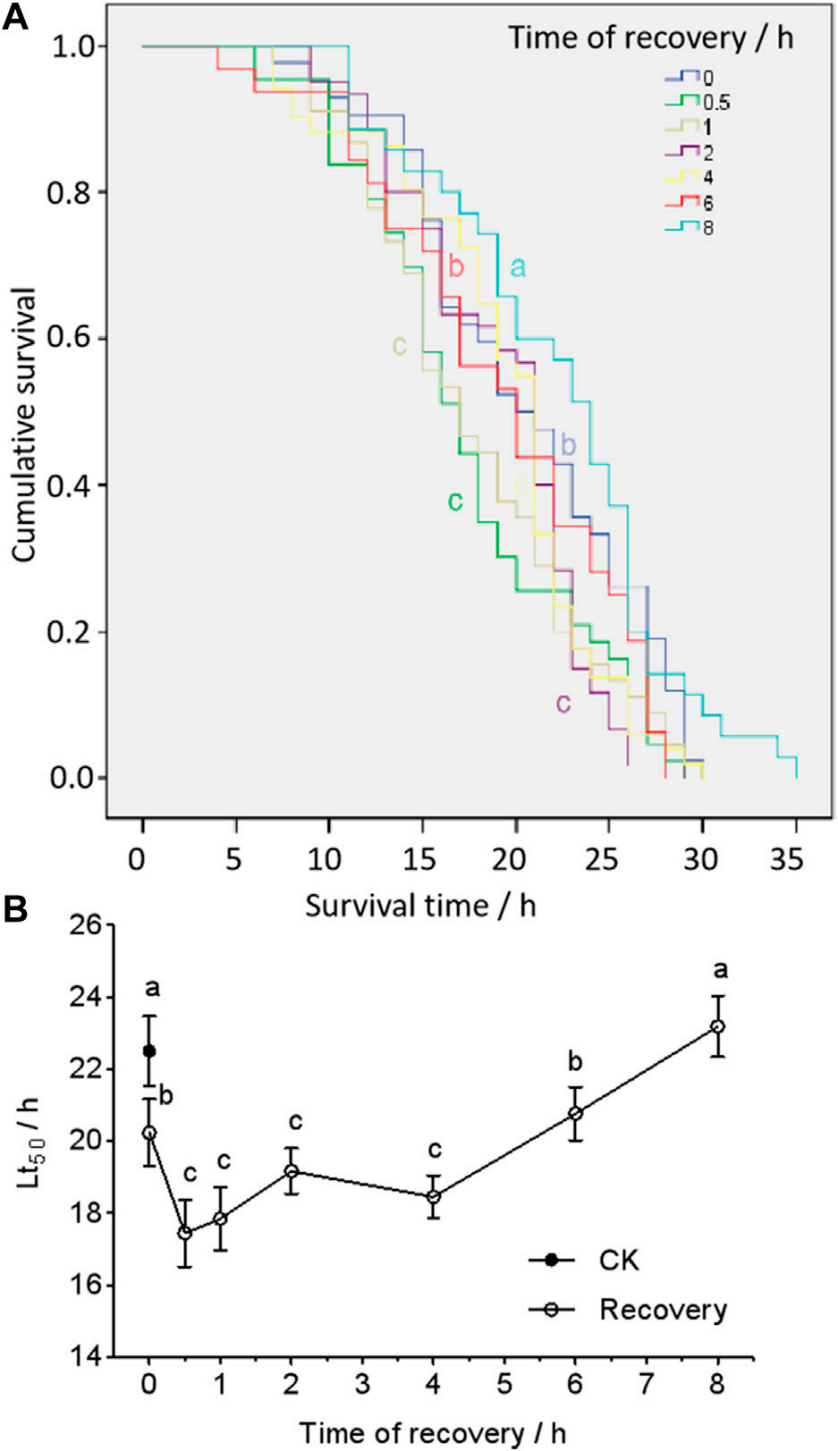
Cumulative survival curves **(A)** and medium lethal time (Lt_50_) **(B)** of larvae post-heat shock at 43°C, 2 h treatment. The curves **(A)** were analyzed by a Kaplan–Meier analysis (chi-squared test), and Lt_50_s **(B)** were from the Kaplan–Meier calculation. The different letters (the color of the letter stands for the same color curve) represent a significant difference (*p* < 0.05).

The survival curves of different recovery time were significantly different in larvae (*df* = 6; *χ* ^*2*^ = 18.7; *p* < 0.05) (Figure 1A). The median lethal time (Lt_50_) (from the Kaplan–Meier function) of survival in heat-shocked larvae (43 C for 2 h) was significantly lower when they were re-exposed to 43 C within 30 min (0.5 h), but it increased as recovery time increased. This reached untreated levels after 6 h of recovery and got significantly longer hereafter (Figure 1).

### Transcriptional fluctuation of *HSP* genes in larvae exposed to different temperatures

**FIGURE 2:**
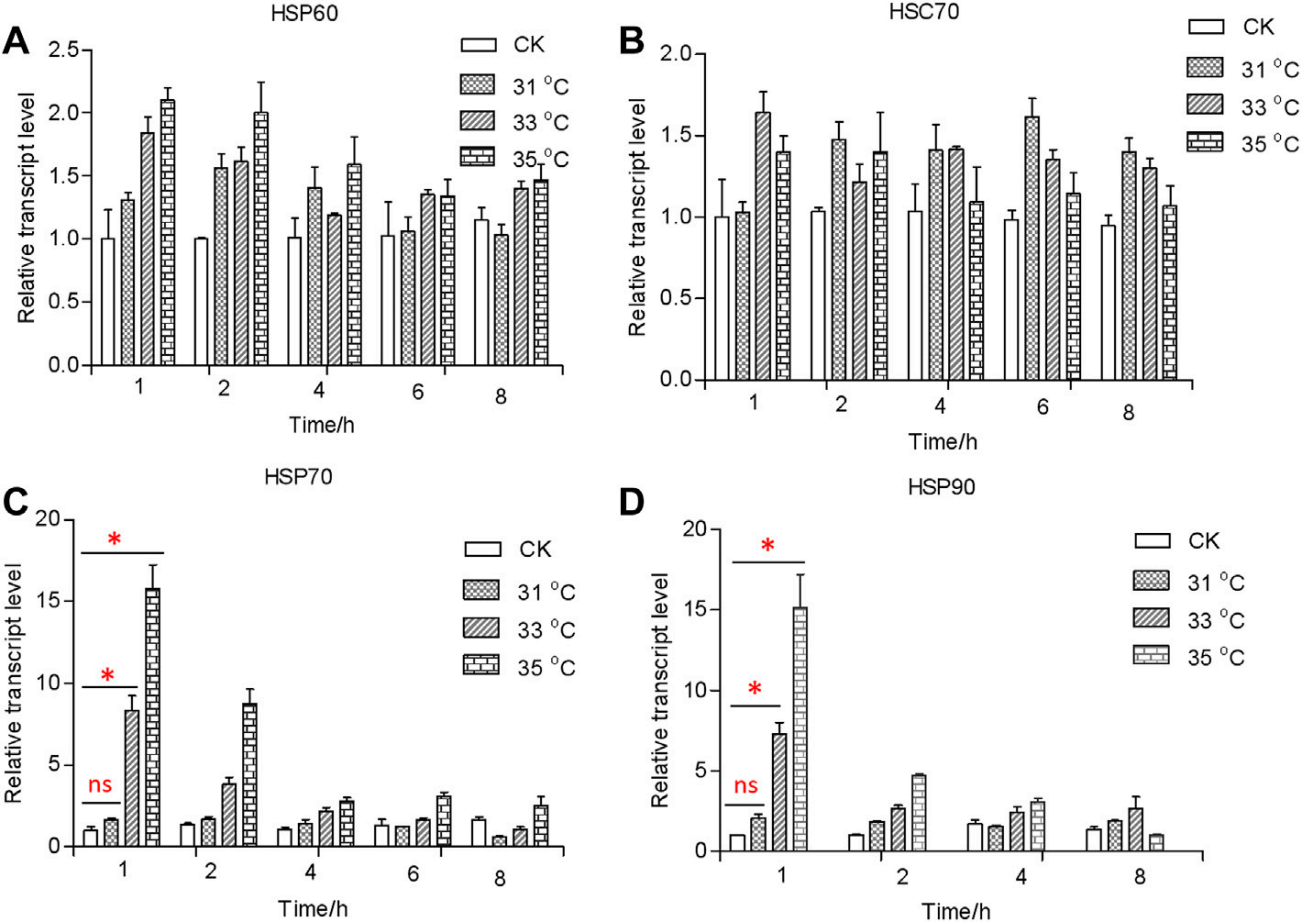
Relative transcript levels of *HSP60* **(A)** *, HSC70* **(B)** *, HSP70* **(C)** *,* and *HSP90* **(D)** under different temperatures. Relative mRNA levels were analyzed using the 2^−∆^^∆CT^ method. All values from two independent replicates are shown as the mean ± SD. The data (*HSP70* and *HSP90*) between CK (27 C) and other treatments (31/33/35 C) at 1 h were analyzed by ANOVA. “*” stands for a significant difference (*p* < 0.05) between CK (27 C) and other treatments; “ns” is not significant.

**FIGURE 3:**
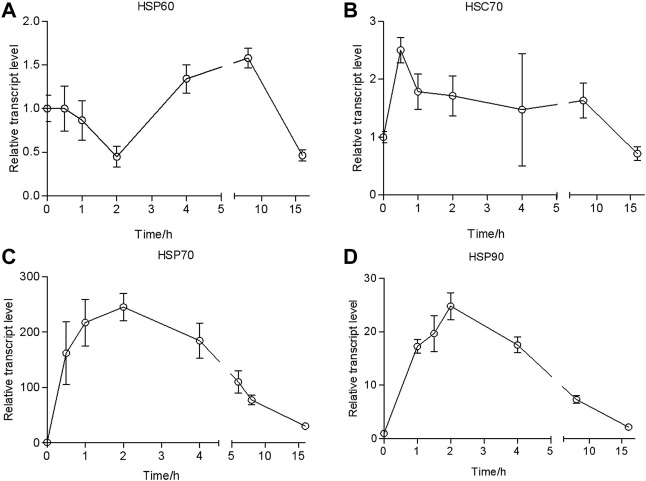
Relative transcript levels of *HSP60* **(A)** *, HSC70* **(B)** *, HSP70* **(C)**, *and HSP90* **(D)** at different times with 43°C treatment. The values from two independent replicates are shown as the mean ± SD, as analyzed by the 2^−△△CT^ method.

In response to the heat shock treatment, the expression levels of the *HSP* genes varied significantly (*F*_*3,20*_ = 699, *p* < 0.001; *F*_*3,20*_ = 581, *p* < 0.001) (Figures 2, 3). In comparison with the control, the transcriptional levels of *HSP*60 and *HSC*70 did not change significantly among all temperature treatments (*F*_*HSP60*_ = 35, *df* = 3, 20, and *p* = 0.073; *F*_*HSC70*_ = 6, *df* = 3, 20, and *p* = 0.364) (Figures 2, 3A,B). By contrast, transcriptional levels of *HSP70* and *HSP90* were significantly upregulated in treatments of exposure to 33 (*F*_*HSP70*_ = 195, *df* = 8, and *p* < 0.05; *F*_*HSP90*_ = 163, *df* = 8, and *p* < 0.05) and 35 C (*F*_*HSP70*_ = 335, *df* = 8, and *p* < 0.001; *F*_*HSP90*_ = 584, *df* = 8, and *p* < 0.364) for 1 h compared to the control exposed at 27°C, but there was no significant difference observed between treatment temperature of 31°C (*F*_*HSP70*_ = 3.1, *df* = 8, *p* = 0.094; *F*_*HSP90*_ = 3.7, *df* = 8, *p* = 0.081) and the control (Figures 2C,D). However, the transcriptional level declined when exposure time increased and was at the untreated level in 4 h. Moreover, *HSP70* could reach a higher peak value at the treatment of 43°C (>10-fold than the 35°C treatment) and remained high longer (16 h) (Figure 3C).

### Dynamics of the transcriptional level of *HSP* genes in heat-shocked larvae

**FIGURE 4:**
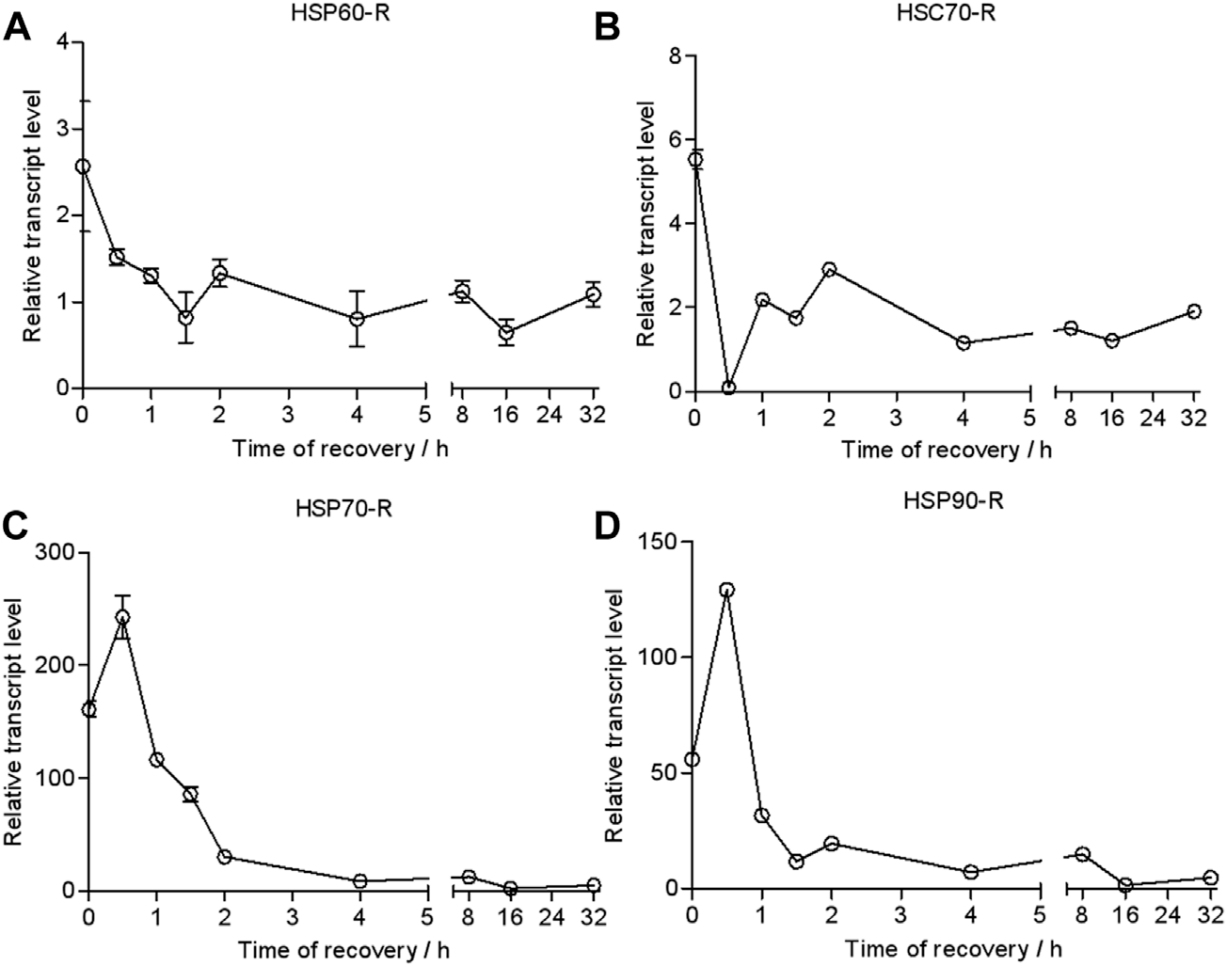
Relative transcript levels of *HSP60* **(A)** *, HSC70* **(B)** *, HSP70* **(C)** *, and HSP90* **(D)** during recovery time (43°C, 2 h treatment). Relative mRNA levels were analyzed using the 2^−∆^^∆CT^ method. The values from two independent replicates are shown as the mean ± SD.

To investigate the responses of HSPs at different recovery times during the thermal tolerance, we quantified the dynamics of transcription of *HSPs* over time. The results of the qRT-PCR analysis showed that the transcriptional level of *HSP60/HSC70* was upregulated only 3- and 5-fold after heat shock compared with the control (Figure 4). It would return to the untreated level after a recovery time of 0.5 h.

By contrast, the transcriptional level of *HSP70* rapidly increased 156-fold compared with the control and reached a peak of 230-fold at a recovery time of 0.5 h. It was dropped to 30-fold at a recovery time of 2 h. It returned to the control level at 16 h (Figure 4).

The transcriptional level of *HSP90* was upregulated 50-fold in response to heat shock. It went up to 140-fold at a recovery time of 0.5 h but dropped down to 30-fold at a recovery time of 1 h. Then, it gradually decreased to the control level in 16 h.

### Effects of recovery time on heat shock-induced transcriptional alteration of HSP genes

**FIGURE 5:**
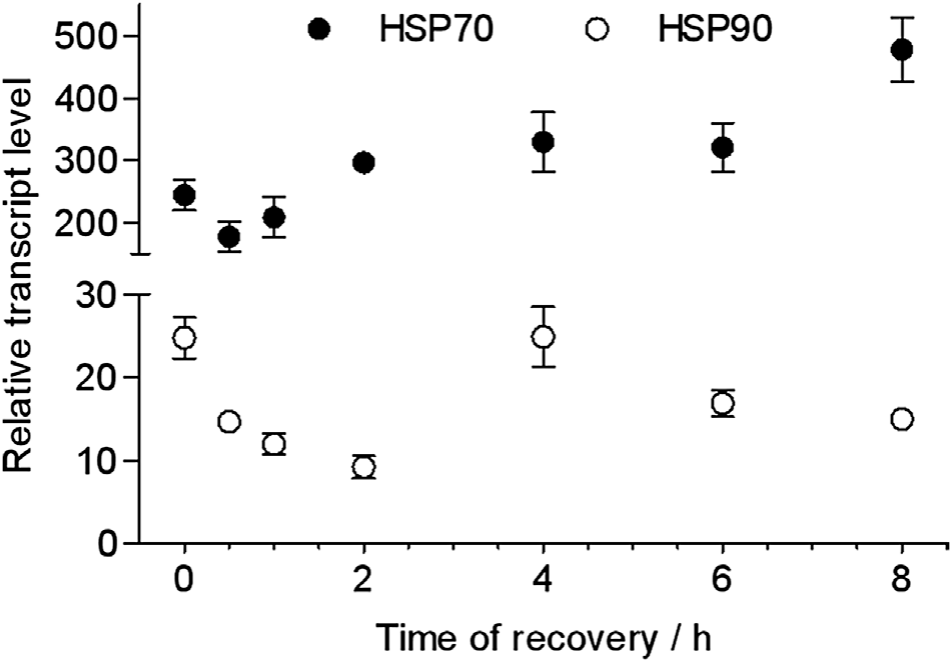
Peak relative transcript levels of *HSP70* and *HSP90* at different recovery times after heat shock at 43 C for 2 h. The values from two independent replicates are shown as the mean ± SD and analyzed by the 2^−△△CT^ method.

The recovery time of heat-shocked larvae significantly influenced the transcription of *HSP70* and *HSP90* genes when they were re-exposed to 43 C (Figure 5). As recovery time increased from 0.5 to 2 h, the peak of the relative transcriptional level of *HSP70* increased from 177- to 209-fold and was lower than the control (0 h recovered, 245-fold) when heat-shocked larvae were resubjected to 43 C for 2 h. However, when recovery time increased to 4–6 h, the relative transcriptional level of *HSP70* was ∼300- and 478-fold (at 8 h). This was similar to *HSP70* whereas the transcription of *HSP90* was upregulated to when 0.5 h recovered larvae were re-exposed to 43 C from 0.5 to 16 h; the peak time was 1 h. However, the transcriptional level was lower than the control (0 h recovered).

## Discussion

Thermal acclimation and/or heat shock can significantly alter thermotolerance in invertebrates, such as *Tribolium castaneum* (Lü and Huo, 2018), *Drosophila melanogaster* (Colinet et al., 2013), and *Nilaparvata lugens* (Piyaphongkul et al., 2014). However, thermal acclimation and/or heat shock-induced heat tolerance varies among the studies due to different exposure regimens, patterns, and hardening. (Dahlgaard et al., 1998; Ju et al., 2011; Piyaphongkul, 2013; Lu et al., 2016; Jin et al., 2019). In this study, the thermotolerance declined within 0.5–4 h after heat shock at 33°C–43°C, but it significantly increased after 6 h. This suggests that post-heat shock time is strongly related to thermotolerance.

Heat shock proteins are expressed as one of the defensive proteins in most organisms in response to various stressful conditions (Feder and Hoffmann, 1999). In most cases, heat-resistant species are characterized by a higher basal level of HSPs than more thermosensitive species. Previous studies showed that increased temperatures elicited activation of a conserved pathway involving heat shock transcription, which enhanced the heat shock response (Rinehart and Denlinger, 2000; Kayukawa et al., 2005; Lopez-Martinez and Denlinger, 2008; Gonsalves et al., 2011; Li et al., 2012; King and MacRae, 2015). In our study, the results showed that temperatures of 31 C–33 C were the threshold triggering *HSP70* gene expression. We also detected increased transcription of *HSP70* and *HSP90* from the high-temperature (35°C–43°C) treatment. The transcription was correlated with the thermal time, but there was no significant change in the transcription of *HSP60* or *HSC70* after heat shock at 35°C–43°C. This suggests that *HSP70* and *HSP90* are responsible for heat-shocked induced defenses. This is similar to that reported for *D. melanogaster*, where expression of HSPs was affected by the heat shock treatment or response (Sørensen et al., 2007).

In addition, the upregulation of *HSP70* or *HSP90* was mostly detected after ∼1–2 h of exposure to heat, then slowed in the following period, and greatly decreased after being transferred to 27 C (for recovery). These results indicated the response to extreme heat events is related to *HSP70* and *HSP90* in *O. furnacalis.* Heat shock proteins are required for survival during heat or cold but cannot maintain a high level of expression in organisms all the time (Scott et al., 1997; Krebs and Feder, 1998; Bowler, 2005). Upregulation of the *HSP70* gene in response to high temperatures was also observed in *Bemisia tabaci* (Hu et al., 2014), *Drosophila buzzatii* (Sørensen et al., 1999), *Anopheles gambiae* (Benoit et al., 2010), and *Nilaparvata lugens* (Lu et al., 2016). The temperatures >31 C upregulate the response of *HSP70* and *HSP90*, but it was not sustainable (for more than 2 h).

The extremely high temperatures can generate damage indirectly by driving an increase in water loss, disrupting the cellular ion balance (hyperkalemia), impairing neurophysiological functions, and damaging mitochondria (O’Sullivan et al., 2017; Bowler, 2018). Insects can produce and accumulate particular molecules to prevent protein denaturation or cell inactivation when they suffer from thermal stimuli (King and MacRae, 2015; Ma et al., 2021). In this study, we observed that the thermal tolerance was significantly lower at the post-heat shock time of 0.5–4 h, with no difference at 6 h, and then enhanced at 8 h. This implies that the insects had internal injuries while suffering heat shock, but it was possibly self-healing within 6 h. Immunological/defensive memory takes over “experience” from the previous injury and rebuilds the immunological/defensive system over 6 h of recovery.

A higher transcription of *HSP70* was detected in a post-heat shock time of 8 h. Correspondingly, the longest median death time was observed at this time. These findings reveal that *HSP70* and *HSP90* are related to the heat shock response and play an important role in heat shock-induced thermotolerance. It also indicates that the inducible HSP70 protects cells or tissue against thermal tolerance and delays thermal injury (Hoffmann et al., 2003; Ruell et al., 2009). Similar findings have been reported in other species. For instance, in *D. melanogaster,* the heat tolerance, metabolic rate, and gene expression significantly change after heat pretreatment (Sørensen et al., 2005; Malmendal et al., 2006). Apple maggot *Rhagoletis pomonella* expresses HSP increasingly from midday to a peak in the afternoon in summer (Lopez-Martinez and Denlinger, 2008). Locusts *Locusta migratoria* are more heat tolerant at low than at high latitudes as a result of their expression pattern of *HSP70* and *HSP90* (Chen et al., 2015b). HSPs (Feder et al., 1997; Lopez-Martinez and Denlinger, 2008) and cuticle proteins (Nguyen et al., 2009) are induced and/or accumulated to deal with extremely high temperatures in *Aphis gossypii* and sorbitol in *Bemisia argentifolii*. In this study, enhancing heat resistance could induce recovery in 6–8 h and is mostly regulated by *HSP70*.

In conclusion, our results suggested that *HSP70* and *HSP90* in *O. furnacalis* are immediately induced by heat events, but *HSP60* and *HSC70* are not. The highest upregulation of *HSP*s is achieved in 2 h and then returns to a normal level at 16 h. In addition, with a post-heat shock time of 0.5–4 h (i.e., recovery time), there is lower thermotolerance, possibly a defensive rebuilding time. Heat-induced thermotolerance achieves at a post-heat shock time of >6 h. Extreme thermal stress-induced quick dynamics of *HSP70* or *HSP90* could be interpreted as an 7adaptation to the drastic and rapid temperature variations. These findings are helpful to understand insect responses to stress.

## Data Availability

The datasets presented in this study can be found in online repositories. The names of the repository/repositories and accession number(s) can be found at: https://www.ncbi.nlm.nih.gov/nuccore/XM_028317890.1, XM_028317890.1; https://www.ncbi.nlm.nih.gov/nuccore/JF708083.1, JF708083.1; https://www.ncbi.nlm.nih.gov/nuccore/XM_028322992.1, XM_028322992.1; https://www.ncbi.nlm.nih.gov/nuccore/GU230734.1, GU230734.1.
