# Supplementary material for: Transcription dynamics of heat shock proteins in response to thermal acclimation in Ostrinia furnacalis
Source: Front Physiol. 2022 Sep 26;13:992293. doi: 10.3389/fphys.2022.992293 (PMC9548879; doi:10.3389/fphys.2022.992293)
Supplement: Supplementary file 1 [file Table1.DOCX]

**Supplementary Material Presentation**

Table S.1 RT-PCR primers of HSP genes and inner reference 18s.

| Primers^*^ | Sequence (5’-3’) |
| --- | --- |
| 18s-R | GACCAGTGATGGGACGAG |
| 18s-F | CTGCCTTCCTTGGATGTG |
| HSP90-R | AGGAAGGCTTGGAGTTGC |
| HSP90-F | ACCGTATTGGGCTGTGA |
| HSP70-R | ATTCCTGCGTCGGAGTTT |
| HSP70-F | AGGTAGGCTTCGGCTGTT |
| HSC70-R | CGTCAGCGATGGAGGCA |
| HSC70-F | CGCAGCAGTCGGTTCGT |
| HSP60-R | ATCAAATCCAGGAAACCACC |
| HSP60-F | CTGAGGGATGTCGCAGATGA |

“*” F: forward, R: reverse.
